# The neuromuscular responses in patients with Parkinson’s disease under different conditions during whole-body vibration training

**DOI:** 10.1186/s12906-021-03481-1

**Published:** 2022-01-03

**Authors:** Chia-Ming Chang, Chon-Haw Tsai, Ming-Kuei Lu, Hsin-Chun Tseng, Grace Lu, Bey-Ling Liu, Hsiu-Chen Lin

**Affiliations:** 1 Department of Physical Therapy, China Medical University, No. 100, Sec. 1, Jingmao Rd, Taichung, Taiwan 406040 R.O.C; 2 Neuroscience Laboratory, Department of Neurology, China Medical University Hospital, Taichung, Taiwan; 3 Division of Parkinson’s Disease and Movement Disorders, Department of Neurology, China Medical University Hospital, Taichung, Taiwan; 4 School of Medicine, College of Medicine, China Medical University, Taichung, Taiwan; 5 Neuroscience and Brain Disease Center, College of Medicine, China Medical University, Taichung, Taiwan; 6 Graduate Institute of Biomedical Sciences, China Medical University, Taichung, Taiwan

**Keywords:** Parkinson’s disease, Whole-body vibration, Knee position, Mechanical vibration frequency, Muscle activation, Acceleration transmissibility

## Abstract

**Background:**

Whole-body vibration (WBV) training can provoke reactive muscle response and thus exert beneficial effects in various neurological patients. This study aimed to investigate the muscles activation and acceleration transmissibility of the lower extremity to try to understand the neuromuscular control in the Parkinson’s disease (PD) patients under different conditions of the WBV training, including position and frequency.

**Methods:**

Sixteen PD patients and sixteen controls were enrolled. Each of them would receive two WBV training sessions with 3 and 20 Hz mechanical vibration in separated days. In each session, they were asked to stand on the WBV machine with straight and then bended knee joint positions, while the vibration stimulation was delivered or not. The electromyographic (EMG) signals and the segmental acceleration from the lower extremity were recorded and processed. The amplitude, co-contraction indexes (CCI), and normalized median frequency slope (NMFS) from the EMG signals, and the acceleration transmissibility were calculated.

**Results:**

The results showed larger rectus femoris (RF) amplitudes under 3 Hz vibration than those in 20 Hz and no vibration conditions; larger tibialis anterior (TA) in 20 Hz than in no vibration; larger gastrocnemius (GAS) in 20 Hz than in 3 Hz and no vibration. These results indicated that different vibration frequencies mainly induced reactive responses in different muscles, by showing higher activation of the knee extensors in 3 Hz and of the lower leg muscles in 20 Hz condition, respectively. Comparing between groups, the PD patients reacted to the WBV stimulation by showing larger muscle activations in hamstring (HAM), TA and GAS, and smaller CCI in thigh than those in the controls. In bended knee, it demonstrated a higher RF amplitude and a steeper NMFS but smaller HAM activations than in straight knee position. The higher acceleration transmissibility was found in the control group, in the straight knee position and in the 3 Hz vibration conditions.

**Conclusion:**

The PD patients demonstrated altered neuromuscular control compared with the controls in responding to the WBV stimulations, with generally higher EMG amplitude of lower extremity muscles. For designing WBV strengthening protocol in the PD population, the 3 Hz with straight or flexed knee protocol was recommended to recruit more thigh muscles; the bended knee position with 20 Hz vibration was for the shank muscles.

## Introduction

Parkinson’s disease (PD) is a neurological degenerative disease, and the patients usually demonstrated rigidity, tremor and bradykinesia. The functional impairments of these patients were observed and affected their balance performance and the ambulation ability (1–3). The difficulty in gait initiation or termination and freezing gait with the increased co-contraction of leg muscles were identified in the PD patients (4). The co-contraction of the agonist and antagonist of lower leg muscles usually presented in the stance phase of gait cycle and also with smaller maximal electromyography (EMG) amplitudes than in the healthy controls (5–7). Moreover, the extensors of the knee joint were found to be weak and easily fatigue in the PD patients (1, 8). The above impairments of musculoskeletal function and inappropriate neuromuscular control in the PD patients are involved with a high incidence of falling (9–13).

For improving the locomotion function and decreasing the risk of falling, exercise intervention is strongly recommended for the PD patients. According to Archibald’s review, a general exercise program was helpful to improve the physical capacity, and a specific exercise intervention could be designed to enhance the balance function of the PD patients (14). Earhart et al. demonstrated that the high-intensity strengthening exercise for the PD patients was beneficial in their force production, muscular endurance and the ambulation performance (15). Furthermore, literature also showed that higher levels of moderate to vigorous physical activities and exercise would not only be associated with better cognitive ability, but also have a neuroprotection effect in PD patients (16, 17). Thus, the exercise including strengthening and endurance training should be highly encouraged for the PD patients to improve cognition, functional ability and prevent falling.

Whole-body vibration (WBV) training, which transmits mechanical vibration to the body via a vibrating platform and thus induces the muscles to contract or relax reactively, has been used for the strengthening and balance training in athletes or healthy adults (18, 19) and also in various clinical patients (20–23). The strengthening effects of using WBV have been investigated with various training conditions, including position and combing with movements (24). The WBV training with slight knee flexion had been reported having better strength enhancement than with the straight knee in the older adults (25). The evaluation of the acceleration transmissibility, which was calculated by the amplitudes of power spectral density from the accelerations on the thigh and the shank, also demonstrated larger substantial vibration to the thigh under the WBV stimulation with slight knee flexion than with the straight knee position in young healthy adults (26). Considering combing movements during WBV training, i.e. static and dynamic squat, Di Iorio et al. showed that static squat would recruit more muscle fibers than the dynamic squat (24). Based on these evidences, the WBV training in static squat could bring supplementary challenges to the lower limb muscles in healthy adults.

The vibration frequency of a WBV machine can be varied from 3 to 500 Hz. Cardinale and Lim reported that the WBV training under 30 Hz could evoke more muscle firing of the vastus lateralis comparing with 40 and 50 Hz in the young women volleyball players (27). Petit et al. presented that the strength of knee extensor and flexor could be increased with a 6-week WBV training under 30 Hz in the young physical active men (28). Despite these studies in young adults showed the WBV around 30 Hz maybe the optimal setting for strengthening the knee extensors, the results may not be generalized to the PD patients. In the literature, the mechanical vibration frequency of the WBV training in PD patients could be divided into the low (3-10 Hz) and high (more than 20 Hz) frequency (29). In regard to the movement disorders in the PD patients that would exhibit with certain frequencies of movement. For example, the resting tremor in PD patients was mostly observed at around 5.5 Hz (30). In addition, the ratio of the frequency power spectrum above 3 Hz to below in the acceleration of the lower leg during walking was used to judge the occurrence of freezing gait in PD patients (31). Therefore, the WBV training using the frequencies higher than 3 Hz, which might reproduce the similar situation of resting tremor or freezing gait, should be considered carefully in the PD patients. However, the comparison of the training effects between low and high WBV frequencies in the PD patients has seldom been discussed.

Based on the above review, the WBV training with high frequency under the static knee flexion showed benefits to the motor fiber recruitment and muscle strength in various populations (27, 28). However, considering the alternations of muscle firing and neuromuscular control in the PD patients, the effect of the WBV training may not be generalized from the previous studies on different population. Therefore, the purpose of this study was to investigate the muscles activation and acceleration transmissibility of the lower extremity in order to understand the neuromuscular control in the PD patients under different conditions of the WBV training, including position and frequency. Furthermore, the results of this study could provide references for the future application of WBV in the PD patients. The hypothesis of this study was that the PD patients would demonstrate different pattern of muscle activation and neuromuscular control compared with healthy population in response to the different conditions in the WBV training.

## Material and methods

### Participants

Sixteen patients with PD were recruited from the Neurology Department of the China Medical University Hospital. The inclusion criteria for the PD group included those who: (1) diagnosed the Parkinson’s disease by the neurology doctor over a year and aged from fifty to eighty; (2) Hoehn and Yahr scale was at level II or III; (3) could stand for 10 min independently. The exclusion criteria were (1) has any other neuromuscular problems; (2) the contraindications of WBV, i.e. server lower limb osteoarthritis or cardiovascular disease; (3) refused to sign a consent form to participate in the study. Another sixteen healthy participants with similar range in age of the PD group were also recruited from the community as the control group. The demographic data of PD and control group was presented in Table 1.

**Table 1:** Demographic data of control and Parkinson groups (mean ± SD)

	Control (*n* = 16)	Parkinson (*n* = 16)	*p*-value
Age (years old)	65.42 ± 5.27	65.12 ± 8.28	0.902
Gender (M:F)	5:11	9:6	0.143^a^
Body height (cm)	158.87 ± 6.43	161.31 ± 7.73	0.339
Body mass (kg)	58.22 ± 8.52	58.94 ± 9.62	0.824
Hoehn and Yahr scale	–	2.50 ± 0.95	–

^a^: Chi-square test

### Instruments

Two WBV machines were used to provide the mechanical vibration frequencies of 3 Hz (i-Vib5050, Real Masters of Health, Taiwan) and 20 Hz (Zen PRO TVR 3900, Jhen-Zan Enterprise, Taiwan) in our study. The Trigno wireless sensors (Delsys, USA) were used to record the EMG signals of selected muscles (sampling rate at 1000 Hz) and the accelerations of the shank and thigh segments (sampling rate at 100 Hz).

### Procedure

**Fig. 1:**
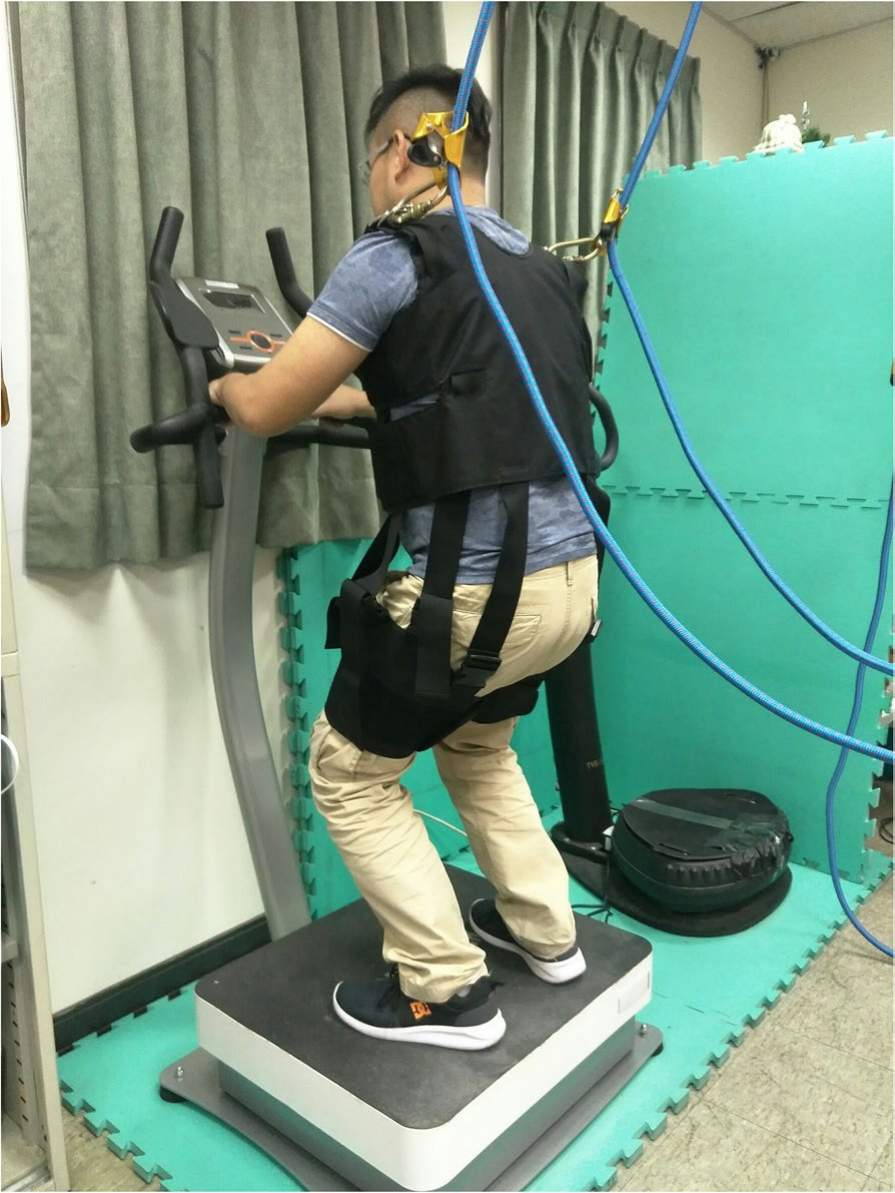
The safety harness and suspension system during the WBV training

All participants were informed fully regarding the experimental procedure and then signed the consent form, which was approved by the Institutional Review Board of China Medical University Hospital (CMUH103-REC3–114), before enrolled. The participants could take their prescription medicines as usual to have better motor function to complete the WBV program. The Trigno wireless sensors were firstly attached at the bilateral rectus femoris (RF), hamstring (HAM), tibialis anterior (TA) and lateral gastrocnemius (GAS), and the maximum voluntary contraction (MVC) tests of these muscles were performed respectively. Then, the participants would be randomly assigned to stand on either one of the two WBV machines for a 5.5-min WBV session and the same testing on another WBV machine with different frequency would be arranged in a few days later. Each session consisted of two standing positions, the straight knees or with knee flexed at 15 degrees, and the participants were asked to maintain each position for 1 min. They would stand first with no vibration (the machine in idle, 0 Hz) and then with the mechanical vibration (start the machine, 3 or 20 Hz). At least 30-s resting interval was given between every one-minute testing condition. The knee angle was confirmed by a goniometer before the testing started. For ensuring the safety of the participant during the WBV session, a harness and suspension system were used with the minimum interference (Fig. 1).

### Data analysis

All EMG signals were processed first with a 20 ~ 400 Hz band-pass filter (4th order Butter). An additional high-pass filter with 50 Hz cut-off frequency was used for removing movement artifacts during the WBV training (32) and a band-stop filter was designed with the selected vibration frequency, 3 or 20 Hz, ±1.5 Hz, for diminishing the signal from the specific WBV frequency (33). The filtered EMG signals were further analyzed using a root-mean-square moving window of 0.01 s with 0.005 s overlapped. Then the linear envelope was accomplished by a 6 Hz low-pass filter. The maximal values during each MVC test of the selected muscles were extracted, and then the processed EMG data during each testing condition would be normalized with their corresponding MVCs. These normalized EMG data were further calculated to obtain the following variables: 1) the amplitude at the middle (50%) of the training time (EMG_50_); 2) the average co-contraction index (CCI) at the 10, 50 and 90% training time of the thigh and shank; 3) the normalized median frequency slope (NMFS) for muscle fatigue trend.

The equation of CCI was as below, function (1) (34).

1 $$\mathrm{CCI}=\frac{\mathrm{EMG}\ \mathrm{amplitude}\ \mathrm{of}\ \mathrm{flexor}}{\mathrm{EMG}\ \mathrm{amplitude}\ \mathrm{of}\ \mathrm{flexor}+\mathrm{EMG}\ \mathrm{amplitude}\ \mathrm{of}\ \mathrm{extensor}}\times 100\%$$

**Fig. 2:**
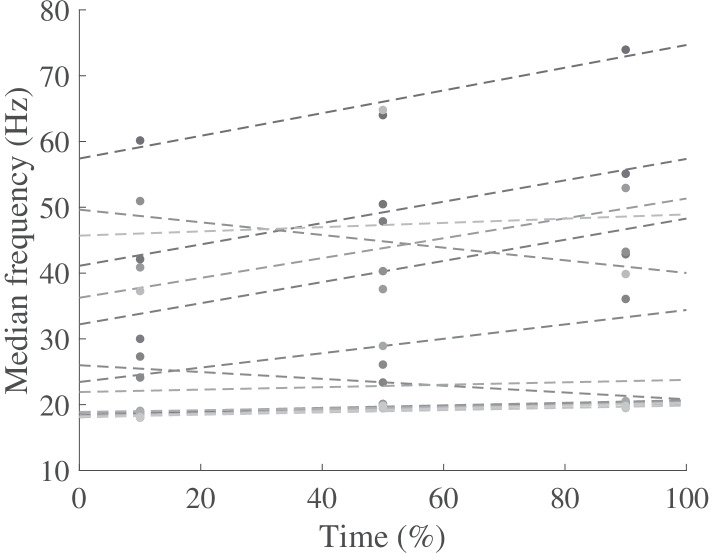
Examples of linear regression for the normalized median frequency slope (dot: the median frequency; line: the regression line)

The flexors here were the hamstring and tibialis anterior, and the extensors were the rectus femoris and medial gastrocnemius for the thigh and shank respectively. The NMFS was calculated by the linear regression of the median frequency at 10, 50 and 90% training time (Fig. 2), and then normalized by the median frequency at 10% (35).

The acceleration data of thigh (a_t_) and shank (a_s_) was retrieved from the Trigno wireless sensors placed on the rectus anterior and tibialis anterior respectively. The acceleration transmissibility was calculated by the transfer function with a moving window of 0.5 s with 0.1 s overlapped [function (2)] (26, 36). The value of the transfer function during 10 to 90% of each testing condition was averaged to represent the performance of the acceleration transmissibility from the shank to the thigh.

2 $$\mathrm{Acceleration}\ \mathrm{transmissibility}=10{\log}_{10}\frac{{\mathrm{PSD}}_{\mathrm{Thigh}}}{{\mathrm{PSD}}_{\mathrm{Shank}}}$$

Where the PSD_Thigh_ was the power spectral densities (PSD) of a_t_ in the ±1 Hz of mechanical vibration frequency using a Hamming window and the PSD_Shank_ was for the a_s_.

### Statistical analysis

The demographic data between groups were compared by the independent t-test for the continuous variables and Chi-square test for gender. A three-way mixed model analysis of variance (ANOVA) (group * frequency * knee joint position) with post hoc test was used to analyze the main effect and interactions in the dependent variables of the EMG and acceleration data. All statistical analyses were calculated using Statistical Package for the Social Sciences software (SPSS, Chicago, USA), and the significance level was set at 0.05.

## Results

The demographic data of two groups was shown in Table 1, and there was no significant difference between groups. The comparisons of EMG_50_ demonstrated significant main effects between groups in the HAM, TA and GAS; between knee positions in the RF and HAM; and among vibration frequencies in the RF, TA and GAS. (Table 2). The post hoc tests for the vibration frequencies indicated following results: 1) RF activation in 3 Hz was larger than in 0 and 20 Hz; 2) TA activation in 20 Hz was larger than in 0 Hz; 3) GAS activation in 20 Hz was larger than in 0 Hz and 3 Hz.

**Table 2:** EMG amplitude at 50% of training time (EMG_50_) in the WBV training under different conditions

		Rectus femoris (% MVC)		Hamstring (% MVC)		Tibialis anterior (% MVC)		Gastrocnemius (% MVC)	
		mean ± SD	*p*-value	mean ± SD	*p*-value	mean ± SD	*p*-value	mean ± SD	*p*-value
Group	Control	10.59 ± 16.06	0.137	4.11 ± 2.98	0.040*	4.07 ± 2.95	0.001*	21.03 ± 18.46	0.004*
	Parkinson	13.41 ± 18.53		8.70 ± 10.29		5.67 ± 5.47		31.71 ± 28.86	
Knee position	Straight	9.51 ± 15.94	0.001*	7.02 ± 8.45	0.042*	4.62 ± 5.30	0.084	27.75 ± 24.94	0.058
	Bended	14.49 ± 18.40		5.78 ± 7.29		5.12 ± 3.43		25.00 ± 24.61	
Vibration frequency	0 Hz	6.33 ± 5.08	< 0.001*	5.80 ± 7.32	0.813	3.44 ± 1.78	0.023*	15.23 ± 10.28	< 0.001*
	3 Hz	20.29 ± 26.09		6.44 ± 5.71		4.72 ± 3.35		21.26 ± 14.68	
	20 Hz	9.38 ± 9.83		6.97 ± 10.11		6.44 ± 6.42		42.62 ± 33.39	

*: *p* < 0.05

The average CCI of thigh were significant larger in the control group than those in the Parkinson group (Table 3). The average CCI of shank had significant interaction (*p* = 0.047) between group and knee position, but the post hoc test did not demonstrate the significant difference (*p* = 0.177). For the NMFS, RF demonstrated the steeper slope under bend knee position than straight knee (Table 4). The NMFS of HAM had main effects in the vibration frequencies. After the post hoc comparisons by the ANOVA, it revealed that the slope was larger in 20 Hz than 3 Hz (*p* = 0.022).

**Table 3:** The average co-contraction index (CCI) in different conditions of the WBV training

		Thigh (%)		Shank (%)^†^	
		mean ± SD	*p*-value	mean ± SD	*p*-value
Group	Control	52.07 ± 11.36	0.049*	22.93 ± 12.32	0.111
	Parkinson	49.69 ± 15.67		24.07 ± 14.93	
Knee position	Straight	51.58 ± 14.49	0.937	21.94 ± 13.60	0.053
	Bended	50.21 ± 12.89		25.07 ± 13.62	
Vibration frequency	0 Hz	48.68 ± 11.87	0.421	26.51 ± 13.25	0.506
	3 Hz	51.48 ± 14.16		24.40 ± 14.25	
	20 Hz	52.54 ± 14.82		19.60 ± 12.72	

*: *p* < 0.05

**Table 4:** Normalized median frequency slope (NMFS) in different conditions of the WBV training

		Rectus femoris (%)		Hamstring (%)		Tibialis anterior (%)		Gastrocnemius (%)	
		mean ± SD	*p*-value	mean ± SD	*p*-value	mean ± SD	*p*-value	mean ± SD	*p*-value
Group	Control	0.15 ± 0.06	0.238	0.18 ± 0.06	0.287	0.33 ± 0.11	0.465	0.26 ± 0.12	0.931
	Parkinson	0.26 ± 0.06		0.09 ± 0.06		0.22 ± 0.11		0.24 ± 0.12	
Knee position	Straight	0.06 ± 0.06	0.010*	0.22 ± 0.07	0.089	0.21 ± 0.10	0.425	0.20 ± 0.06	0.568
	Bended	0.34 ± 0.08		0.06 ± 0.05		0.34 ± 0.12		0.30 ± 0.16	
Vibration frequency	0 Hz	0.26 ± 0.09	0.353	0.18 ± 0.08	0.030*	0.14 ± 0.07	0.458	0.23 ± 0.08	0.553
	3 Hz	0.10 ± 0.09		−0.01 ± 0.05		0.37 ± 0.19		0.35 ± 0.23	
	20 Hz	0.25 ± 0.07		0.25 ± 0.08		0.32 ± 0.12		0.17 ± 0.06	

*: *p* < 0.05

**Fig. 3:**
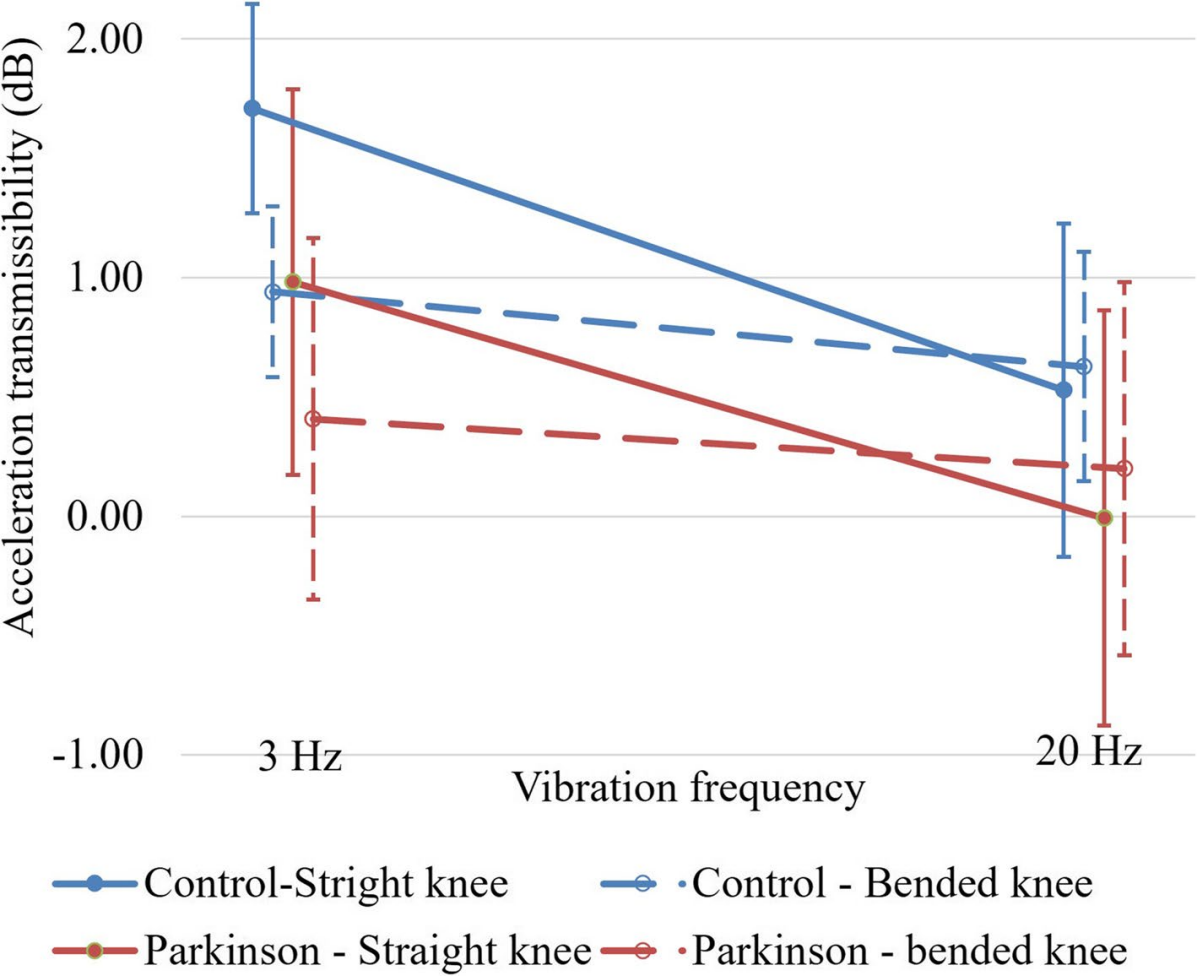
The interaction of acceleration transmissibility (blue line: the control group, red line: the Parkinson group; solid line: straight knee, dash line: bended knee)

All main effects were significant for the acceleration transmissibility (group: *p* = 0.017, knee joint position: *p* < 0.001 and vibration frequency: *p* < 0.001). The control group has larger acceleration transmissibility than the Parkinson group (Fig. 3, blue line v.s. red line); the straight knee position has larger acceleration transmissibility than the bended knee position (Fig. 3, solid line v.s. dash line); vibration frequency in the 3 Hz has larger acceleration transmissibility than the 20 Hz (Fig. 3, left side v.s. right side). The interaction was showed between the knee position (straight/ bended) and the vibration frequency (3 Hz/ 20 Hz) (*p* < 0.001). Further analysis demonstrated that the acceleration transmissibility under the combination of straight knee and vibration in 3 Hz demonstrated larger acceleration transmissibility than the other conditions: straight knee in 3 Hz (*p* = 0.001), bended knee in 3 Hz (*p* < 0.001) and 20 Hz (*p* < 0.001).

## Discussion

The current study compared the muscle activation and acceleration transmissibility of the lower extremity between the PD patients and the control participants in response to the WBV training under different mechanical vibration frequencies and changed knee joint positions. Our results showed the Parkinson patients demonstrated different muscle activation pattern and neuromuscular control comparing to the control participants in responding the WBV training. These results could provide some evidence to design the intervention for the PD patients using the WBV training.

The results of EMG_50_ indicated that the PD patients would have higher activation in all these lower extremity muscles than the control group, expect for the RF (Table 2). To the comparisons among the vibration frequencies, the 3 Hz mechanical vibration would significantly stimulate the RF and demonstrated the highest EMG_50_ amplitude than other vibration frequencies. The muscle firing which responded to the 20 Hz mechanical vibration was discovered in the lower leg, i.e. both TA and GAS was recruited to adapt the vibration. The major changes between knee joint positions appeared in the thigh muscles. The HAM was demanded in the bended knee position, while the RF in the straight knee position. This result is consistent with the Pollock et al.’s study. Their results demonstrated that straight knee position would evoke more firing of the RF and the lower leg muscles under the high mechanical vibration frequency than the bended knee position (33). For the results of general high EMG amplitude of lower extremity muscles in the PD patients, it may be due to the weak knee extensors. The weakness in the knee extensor in the PD patients was noted and reported in the literature (1, 8). During the WBV training, the participants need to adequately recruit lower extremity muscles to stabilize the joint, control the posture and maintain the balance. Therefore, HAM, TA and GAS may have to increase firing in order to compensate the weak RF and obtain the above function.

For the results of CCIs, the PD patients demonstrated slight lower co-contraction at the thigh (Table 3). Unlike to our results, previous studies showed the small EMG amplitude and high CCI in the shank (5, 6). This mismatched may come from the effect of their prescribed medicine. In current study, we did not ask the PD patients to interrupt their regular medical intervention, so the symptom of rigidity may be controlled by their prescribed medicine. Consequently, the phenomenon of co-contraction between the agonist and antagonist may not be affected by the pathological muscle tension. Moreover, the difference in the thigh CCI between groups was very small, less than 3%, though the *p*-value of this comparison was just reaching the statistically significant level. Thus, this significance between groups may not have clinical meaningfulness, and showing that this type of exercise would not induce abnormal muscle tone in the PD patients.

The NMFS was analyzed to show the trend of the muscle fatigue during the WBV exercise. It demonstrated differences between the knee positions and among the vibration frequencies. Regard to the knee positions, the regression line of median frequency of the RF in the straight knee position demonstrated smaller slope and close to the horizontal axis than in the bended knee position (Table 4). For the HAM, the regression line in the vibration frequency at 3 Hz was also almost parallel with the horizontal axis and showed significant difference compared to NMFS in the 20 Hz condition (Table 4). Although with these significant differences between conditions, the NMFS were overall close to zero. It may reflect that the dosage of our WBV training program would not reach to the fatigue level for all these lower extremity muscles.

As to the acceleration transmissibility, the thigh would receive more stimuli than the lower leg by the mechanical vibration in all conditions and participants, i.e. the values of acceleration transmissibility were all larger than the 0 dB. Our results showed the protocol of the straight knee with vibration in the 3 Hz in the WBV training would demonstrate high acceleration transmissibility, suggesting a large gain of the mechanical vibration stimuli via the knee joint in this condition (Fig. 3). In the previous study, the acceleration transmissibility in healthy young individuals showed all negative values, indicating the trend of mechanical vibration majorly stimulate on the lower leg and was partially absorbed here. The changes of the acceleration transmissibility in different WBV conditions showed more negative with the increasing vibration frequency and in the bended knee position (26). This different trend may come from the different population and experimental setup. In our study, older participants were studied and they were asked to hold the handrail of the WBV machine during intervention for the safety concern. This position may assist the participants to maintain the stability of the upper body but also limit the movability, and thus make the mechanical vibration stimuli mainly on the thigh, turned the acceleration transmissibility to positive value. In addition, previous study also showed the different sign of value in the acceleration transmissibility in different age range of the participants in walking (37), suggesting different neuromuscular control strategy in the older individuals. The results of acceleration transmissibility were echoed with the comparisons of EMG amplitude. When the participants received the 3 Hz mechanical vibration stimuli with the straight knee, these stimuli would concentrate on the thigh and thus induced significantly higher thigh muscles activation (Table 2).

According to the results of this study, the PD patients indeed presented a different neuromuscular control in responding to the WBV intervention showing in the EMG amplitude, performance of the co-contraction, and the acceleration transmissibility. The training protocol of 3 Hz mechanical vibration with the bended knee position may be suggested for the thigh muscles strengthening in both control and PD population, and 20 Hz mechanical vibration may be recommended to train the shank muscles. Considering the safety of the WBV training, our training dosage showed no induced hypertonicity or muscle fatigue, so this protocol could be used with confidence for the PD patients. As the previous study has mentioned, enhancement of muscle strength in the lower extremity could improve the functional performance, especially ambulation, in the PD patients (38–43). Especially, the balance function and falling rate could be improved after the thigh muscles strengthening program (11, 12); the freezing of gait was also related to the strength of shank muscles in the PD patient (5). These functional improvements could be expected by a tailored WBV training program in long term.

In current study, there were several limitations. First was the limited sample size were enrolled, only 16 in each group. Second limitation was the PD patients were tested under the medicine effect due to the safety concern and the preference of participants for completing the WBV exercise session. The final limitation was that we have just evaluated the response in the WBV session but not included the training effect. In current study, we focused only on the muscle recruitment and acceleration between segments during the WBV training. As to the training effect of WBV, there still need further studies.

## Conclusion

The PD patients demonstrated altered neuromuscular control compared with the controls in responding to the WBV stimulations, with generally higher EMG amplitude of lower extremity muscles. The different conditions of WBV training could be used to encourage specified muscle activation. The 3 Hz WBV with straight or flexed knee protocol was recommended to recruit more thigh muscles; the bended knee position with 20 Hz vibration was for the shank muscles. The results of our study could provide some reference for designing strengthening protocol in the PD population using the WBV machine, and further study with the long-term intervention should be designed for evaluating the effect of strengthening by the WBV exercise.

## Data Availability

The datasets used and/or analyzed during the current study are available from the corresponding author on reasonable request.

## Abbreviations

ANOVA: Analysis of variance
CCI: Co-contraction index
GAS: Gastrocnemius
HAM: Hamstring
MVC: Maximum voluntary contraction
NMFS: Normalized median frequency slope
PD: Parkinson’s disease
PSD: Power spectral densities
RF: Rectus femoris
TA: Tibialis anterior
WBV: Whole-body vibration
